# West Syndrome in an Infant With Vitamin B12 Deficiency Born to Autoantibodies Positive Mother

**DOI:** 10.3389/fped.2019.00531

**Published:** 2019-12-20

**Authors:** Pin Fee Chong, Masaru Matsukura, Kaoru Fukui, Yoriko Watanabe, Naomichi Matsumoto, Ryutaro Kira

**Affiliations:** ^1^Department of Pediatric Neurology, Fukuoka Children's Hospital, Fukuoka, Japan; ^2^Department of Pediatrics and Child Health, Kurume University School of Medicine, Kurume, Japan; ^3^Research Institute of Medical Mass Spectrometry, Kurume University School of Medicine, Kurume, Japan; ^4^Department of Human Genetics, Yokohama City University Graduate School of Medicine, Yokohama, Japan

**Keywords:** west syndrome, infantile spasm, cobalamin, anti-intrinsic factor antibody, anti-parietal cell antibody

## Abstract

Infantile vitamin B12 deficiency, a rare nutritional disorder in developed countries, is characterized by megaloblastic anemia and non-specific symptoms, including failure to thrive, hypotonia, and seizure. Symptoms usually develop at 6 months of age. Exclusively breast-fed infants of vegan-diet mothers are particularly at risk. We report the case of a 7-month-old boy with West syndrome born to a mother with subclinical vitamin B12 deficiency due to autoantibodies. Electroencephalography revealed the characteristic hypsarrhythmia pattern, whereas cranial magnetic resonance imaging revealed cerebral atrophy and hypomyelination. Biochemical analysis revealed elevated urinary methylmalonic acid and homocysteine and reduced plasma methionine. Serum vitamin B12 levels were extremely low in both the child and his mother. The mother tested positive for both anti-intrinsic factor and anti-parietal cell antibodies. Low-dose adrenocorticotropic hormone was effective for seizure control. Contrary to previous reports, age-appropriate neurodevelopment was not achieved despite rapid normalization of metabolic profile with vitamin B12 supplementation. Further investigations failed to detect any causative mutations in the genes associated with developmental and epileptic encephalopathy as well as metabolic and other identifiable disorders known to cause West syndrome. To the best of our knowledge, this is the first reported case in which maternal anti-intrinsic factor antibody was considered to be the reason for infantile vitamin B12 deficiency with West syndrome. Differential diagnosis of West syndrome should include vitamin B12 deficiency due to its treatable nature, and early diagnosis is essential to prevent permanent neurological consequences.

## Background

West syndrome, an age-related epileptic disorder affecting children during infancy and early childhood, is characterized by epileptic spasms occurring in clusters and prominent interictal epileptiform discharges (1). Children with West syndrome have a high risk of developing cognitive deterioration, thereby warranting early and aggressive anti-epileptic treatment. Many conditions are associated with this heterogeneous disorder, including early brain insults, malformations, chromosomal anomalies, inborn errors of metabolism, and mutations or genomic deletions in disease-associated genes (1). Despite its rarity, early diagnosis of metabolic disorders, such as phenylketonuria, Menkes disease, and pyridoxine deficiency, is important since it may result in a specific treatment (2).

Herein, we describe a case of West syndrome in an exclusively breast-fed infant with secondary dietary vitamin B12 deficiency due to subclinical maternal deficiency. The mother had significant vitamin B12 deficiency despite normal maternal diets. Further investigations revealed the presence of anti-intrinsic factor antibody (AIFA) and anti-parietal cell antibody (APCA) as the underlying etiology.

## Case Presentation

**Figure 1:**
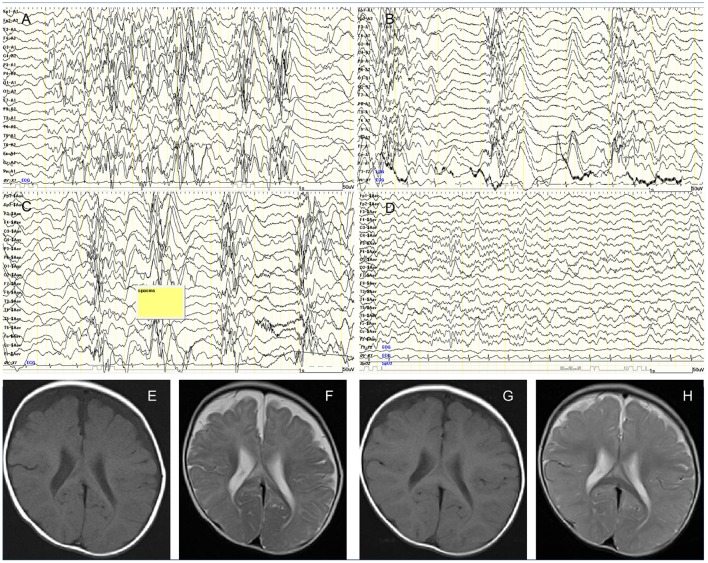
**(A)** Interictal awake electroencephalography at 7 months showing the characteristic random high-voltage slow waves with spikes and polyspikes activity. **(B)** Fragmentation of the hypsarrhythmic activity is noticed during sleep recording. **(C)** Ictal EEG recording showing a cluster of epileptic spasms with high amplitude slow wave at each spasm. **(D)** Sleep recording showed normalization of background EEG observed 2 months after treatment. **(E)** Axial T1-weighted image. **(F)** Axial T2-weighted image of cranial MRI before initiation of adrenocorticotropic hormone therapy at 7 months of age showing cerebral atrophy with frontal predominance and delay in myelination. Follow-up imaging after vitamin B12 supplementation at 14 months of age documenting improved delayed myelination and cerebral atrophy. **(G)** Axial T1-weighted image. **(H)** Axial T2-weighted image.

A 7-month-old boy was born at full term after an uneventful pregnancy of 39 weeks. His birth weight was 2,708 g (−1.1 standard deviation scores, SDS) and head circumference was 33.8 cm (0.4 SDS). Expanded newborn screening, including tandem mass spectrometry, detected no abnormalities. He is the second child of healthy non-consanguineous parents, and their family medical history is unremarkable. At 5 months, the patient had missed developmental milestones and became hyporesponsive with decreased general activity. At 7 months of age, he had recurrent episodes of head nodding with sudden extension of the extremities and upward eye deviations occurring in clusters. Although exclusively breast-fed, he showed no other symptoms of malnutrition with proper weight gain and healthy skin. Interictal electroencephalograhy revealed typical hypsarrhythmia patterns during sleep and awake (Figures 1A,B). Ictal EEG demonstrated the typical findings of epileptic spasms (Figure 1C). Head circumference was 43.7 cm (−0.2 SDS), and cranial magnetic resonance imaging (MRI) revealed cerebral atrophy and delayed myelination (Figures 1E,F). Blood count analysis showed non-macrocytic anemia, whereas metabolic analysis documented methylmalonic aciduria, homocystinuria, and low serum methionine, suggesting vitamin B12 deficiency (Table 1). Serum vitamin B12 level of <100 pg/mL is considered severely deficient (3); the patient's vitamin B12 level (52 pg/ml) was detected to be profoundly low. Therefore, he was diagnosed with West syndrome associated with vitamin B12 deficiency.

**Table 1:** Admission and follow-up biochemical parameters of the patient.

**Parameter**	Reference value^*^	**At diagnosis, 7 months**	**Vitamin B12 supplementation period (months)**				**Last follow-up, 20 months**
			**1**	**2**	**3**	**5**	
Hemoglobin	10.5–14.1 g/dL	9.8	11.4	NT	10.8	10.3	11.8
Hematocrit	32–42%	29.4	33.9	NT	33.8	33.1	36.4
Mean corpuscular volume	72–87 fL	86.5	90.6	NT	81.6	73.9	75.4
Mean corpuscular hemoglobin	23–30 pg	28.8	30.5	NT	26.1	23	24.4
Vitamin B12	180–914 pg/mL	51	>1,500	>1,500	>1,500	>1,500	>1,500
Folic acid	>4.0 ng/mL	>22.0	NT	NT	NT	NT	10.6
Total homocysteine	4–14 nmol/mL	NT	3.6	3.1	3.4	NT	3.2
Methionine	19–41 nmol/mL	8.3	22.2	14.5	14.7	31.8	19.2
Urinary methylmalonic acid	<20 mmol/mol creatinine	347.3	ND	ND	ND	0.9	ND
Urinary 3-OH-priopionic acid	<20 mmol/mol creatinine	68.2	8.2	1.4	4.3	3.0	14.2
Urinary homocysteine	Negative (U/mg creatinine)	0.4	ND	ND	ND	ND	ND
AIFA	Negative	Negative	NT	NT	NT	NT	NT
APCA	Negative	Negative	NT	NT	NT	NT	NT

*AIFA, anti-internal factor antibody; APCA, anti-parietal cell antibody; ND, not detected; NT, not tested*.

* *Normal range value of age-appropriate Japanese population was used*.

While topiramate (maximum dose; 9 mg/kg/day) did not improve the seizure frequency, intramuscular low-dose synthetic adrenocorticotropic hormone (daily dosage of 0.0125 mg/kg for 2 weeks, gradually tapered off to once every other day for 2 weeks, twice weekly for 2 weeks, and then weekly for 2 weeks) was effective for seizure control. Follow-up EEG at 2 months showed normalization of background EEG (Figure 1D). Vitamin B12 replacement therapy was intramuscularly administered to the patient with an initial dose of 1 mg/day. The dose was gradually tapered and subsequently switched to oral supplementation (500 μg/day) after 3 months. The patient became more active and regained social smile after 2 months of supplementation. Although his psychomotor development improved gradually, age-appropriate developmental milestones were not achieved. Denver Development Screening Test revealed that his psychomotor development was as low as 13 months at 20 months of age. Normalization of vitamin B12 level was achieved despite termination of supplementation at 18 months of age.

Normal genetic testing of *MMACHC, MMADHC, LMBRD1*, and *HCFC1* genes ruled out intracellular cobalamin metabolism disorders, which may present similar metabolic profiles (methylmalonic aciduria, homocystinuria) in the absence of vitamin B12 deficiency (4). Neurological comorbidity of delayed psychomotor development despite vitamin B12 supplementation prompted further evaluation for other genetic etiologies of West syndrome. Chromosomal analysis via G-banding revealed normal male karyotype. Whole-exome sequencing was performed as previously described (5), and no causative *de novo* point mutations in previously known developmental and epileptic encephalopathies-associated genes, including *ARX, KCNT1, KCNQ2, SCN1A, SCN2A, SCN8A, STXBP1, SPTAN1, GNAO1, GRIN1, FOXG1, QARS, EEF1A2, PIGA, CDKL5, SLC35A2, CASK, PCDH19*, or *MECP2*, were found. Copy number variants analysis by eXome Hidden Markov Model algorithm detected no pathogenic variant. The ethics committee of Yokohama City University School of Medicine approved the experimental protocols.

The symptom-free mother underwent evaluation for suspected vitamin B12 deficiency. She had a history of iron deficiency anemia and was given iron supplementation during pregnancy. Macrocytosis was present, and serum vitamin B12 level was severely low (85 pg/mL) despite a normal diet (Table 2). Normal esophagogastroduodenoscopy finding and pathological findings confirmed the absence of *Helicobacter pylori*-associated atrophic gastritis. Immunological testing showed positive AIFA and APCA.

**Table 2:** Biochemical parameters of the mother.

**Parameter**	**Reference value**	**At diagnosis**
Hemoglobin	11.6–14.8 g/dL	12.9
Hematocrit	35–44%	38.3
Mean corpuscular volume	84–98 fL	104.4
Mean corpuscular hemoglobin	28–32 pg	35.1
Vitamin B12	180–914 pg/mL	85
Folic acid	>4.0 ng/mL	6.2
Iron	40–188 μg/dL	100
Ferritin	13–301 ng/mL	5.5
Total iron binding capacity	290–335 μg/dL	349
Total homocysteine	4–14 nmol/mL	22.7
Methionine	19–41 nmol/mL	18
Urinary methylmalonic acid	<20 mmol/mol creatinine	ND
Urinary homocysteine	Negative	ND
Antinuclear antibody	<1:40	<1:40
AIFA	Negative	Positive
APCA	<10 Units	20

*AIFA, anti-internal factor antibody; APCA, anti-parietal cell antibody; ND, not detected*.

## Discussion

**Figure 2:**
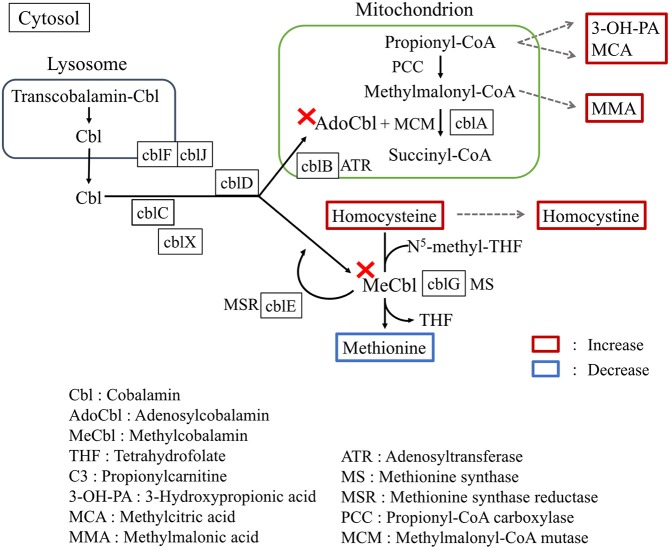
Summarized pathways involving cobalamin metabolism. The map shows the metabolic consequences of vitamin B12 deficiency.

Vitamin B12 (cobalamin) is a water-soluble vitamin mostly found in trace amounts in animal-source foods and acts as an essential cofactor (Figure 2) for conversion of methymalonyl-CoA to succinyl-CoA as well as for methylation of homocysteine to methionine (3). Infantile vitamin B12 deficiency is relatively rare in developed countries, and usually occurs in exclusively breast-fed infants of vegan-diet mothers (6). In our present case, immunologically proven maternal APCA and AIFA are the reasons for vitamin B12 deficiency. Negative detection for both APCA and AIFA at diagnosis rules out the involvement of autoantibodies. Transplacentally acquired AIFA in neonatal cases revealed that the antibody titer significantly decreases in subsequent months and disappears at approximately 3 months (7). The lack of elevated maternal antibody documentation in the child is a limitation of this study.

Hematological manifestations of vitamin B12 deficiency comprises macrocytosis, and in severe cases, megaloblastic anemia (8). Typical but non-specific neurological manifestations in infants include hypotonia, psychomotor retardation or regression, seizures, movement disorders, and failure to thrive (8–10). Active transplacental transport causes 2-times higher cord blood vitamin B12 level than the level in the mother at birth (10), leading to occurrence of symptoms at approximately 6 months after depletion of hepatic reserve. The time of disease manifestation and progression depends on the severity of maternal deficiency.

Epilepsy is a rare clinical manifestation of infantile vitamin B12 deficiency (9), although few reports have described its causal association with infantile spasms including West syndrome (11–14). In general, West syndrome prognosis is unfavorable due to frequent neurological comorbidity; however, all five infants with vitamin B12 deficiency-associated West syndrome showed good prognosis with good seizure outcome, and none showed neurodevelopment delay at the last follow-up (11–14). All children were exclusively breast-fed, and all, except one child, were due to maternal vegetarian diet. Maternal APCA was positive with AIFA undetected in the remaining one child (11).

Delayed myelination, demyelination, axonal degeneration, neurotoxic cytokines imbalance, and accumulation of lactate in the brain cells were hypothesized as the underlying pathophysiological mechanisms for vitamin B12 deficiency in the developing brain (6, 15). Furthermore, the metabolic consequences of homocysteine accumulation and methionine depletion may inflict damages to the central nervous system. Accumulation of homocysteine disrupts the ischemic tolerance by increased oxidated stress and acceleration of atherosclerotic changes (16). A decreased level of methionine causes subsequent low level of S-adenosylmethionine, which functions as a methyl donor acting in a wide variety of biological methylations, resulting in demyelination (17). The initial MRI in our case showed morphological changes of hypomyelination and cerebral atrophy, with further improved MRI findings (Figures 1G,H) possibly due to the treatment. Because brain myelination is most active in the first 6 months of life, vitamin B12 deficiency during this period can inflict profound neurological damage. The low maternal vitamin B12 level might have caused irreversible fetal cerebral impairment and subsequently West syndrome in our case. Since the child presented developmental regression from 5 months, West syndrome might have begun much earlier. This delay in diagnosis might offer another possible explanation for the developmental delay despite treatment.

Differential diagnosis of West syndrome should include both genetically inherited metabolic disorders, and “environmentally” acquired metabolic disorders due to autoantibodies or metabolites from untreated mothers. As vitamin B12 deficiency is treatable, clinicians should maintain a high index of suspicion in exclusively breast-fed children presenting failure to thrive, particularly in cases showing seizure or neurodevelopmental delay. Prompt investigation of the child and mother for any comorbidities is also necessary. In the present case, treatment for iron deficiency anemia during pregnancy might have somehow masked the hematological anomaly, thereby causing diagnostic delay of the maternal condition. Infantile nutritional vitamin B12 deficiency can be eminently treatable; therefore, timely diagnosis is warranted to avert permanent neurological consequences.

## Data Availability Statement

All datasets generated for this study are included in the article/supplementary material.

## Ethics Statement

Written informed parental consent was obtained for publication of this case report.

## Author Contributions

PC and RK conceptualized and designed the study, drafted the initial manuscript, and reviewed and revised the manuscript. MM, KF, and YW performed the initial analyses and reviewed the revised manuscript. NM designed the data collection instruments, supervised data collection, and critically reviewed the manuscript. All authors approved the final manuscript as submitted and agree to be accountable for all aspects of the work.

### Conflict of Interest

The authors declare that the research was conducted in the absence of any commercial or financial relationships that could be construed as a potential conflict of interest.
